# GnRH agonist and hCG (dual trigger) versus hCG trigger for follicular maturation: a systematic review and meta-analysis of randomized trials

**DOI:** 10.1186/s12958-021-00766-5

**Published:** 2021-06-01

**Authors:** Kai-Lun Hu, Siwen Wang, Xiaohang Ye, Dan Zhang, Sarah Hunt

**Affiliations:** 1 Key Laboratory of Reproductive Genetics (Ministry of Education) and Department of Reproductive Endocrinology, Women’s Hospital, School of Medicine, Zhejiang University, Hangzhou, Zhejiang 310006 P. R. China; 2 Key Laboratory of Women’s Reproductive Health of Zhejiang Province, Hangzhou, Zhejiang 310006 P. R. China; 3 Department of Obstetrics and Gynaecology, Monash University, Clayton, Victoria Australia

**Keywords:** GnRH agonist, hCG, Dual trigger, Randomized trial, Systematic review, Meta-analysis

## Abstract

**Background:**

Traditionally, final follicular maturation is triggered by a single bolus of human chorionic gonadotropin (hCG). This acts as a surrogate to the naturally occurring luteinizing hormone (LH) surge to induce luteinization of the granulosa cells, resumption of meiosis and final oocyte maturation. More recently, a bolus of gonadotropin-releasing hormone (GnRH) agonist in combination with hCG (dual trigger) has been suggested as an alternative regimen to achieve final follicular maturation.

**Methods:**

This study was a systematic review and meta-analysis of randomized trials evaluating the effect of dual trigger versus hCG trigger for follicular maturation on pregnancy outcomes in women undergoing in vitro fertilization (IVF). The primary outcome was the live birth rate (LBR) per started cycle.

**Results:**

A total of 1048 participants were included in the analysis, with 519 in the dual trigger group and 529 in the hCG trigger group. Dual trigger treatment was associated with a significantly higher LBR per started cycle compared with the hCG trigger treatment (risk ratio (RR) = 1.37 [1.07, 1.76], I^2^ = 0%, moderate evidence). There was a trend towards an increase in both ongoing pregnancy rate (RR = 1.34 [0.96, 1.89], I^2^ = 0%, low evidence) and implantation rate (RR = 1.31 [0.90, 1.91], I^2^ = 76%, low evidence) with dual trigger treatment compared with hCG trigger treatment. Dual trigger treatment was associated with a significant increase in clinical pregnancy rate (RR = 1.29 [1.10, 1.52], I^2^ = 13%, low evidence), number of oocytes collected (mean difference (MD) = 1.52 [0.59, 2.46), I^2^ = 53%, low evidence), number of mature oocytes collected (MD = 1.01 [0.43, 1.58], I^2^ = 18%, low evidence), number of fertilized oocytes (MD = 0.73 [0.16, 1.30], I^2^ = 7%, low evidence) and significantly more usable embryos (MD = 0.90 [0.42, 1.38], I^2^ = 0%, low evidence).

**Conclusion:**

Dual trigger treatment with GnRH agonist and HCG is associated with an increased live birth rate compared with conventional hCG trigger.

**Trial registration:**

CRD42020204452.

**Supplementary Information:**

The online version contains supplementary material available at 10.1186/s12958-021-00766-5.

## Background

Infertility is a common condition affecting more than 10% of women of reproductive age worldwide [1, 2]. Since the birth of the first IVF conceived baby in 1978, millions of couples have received this treatment which in broad terms includes controlled ovarian hyperstimulation, fertilization in vitro and embryo transfer.

In humans, spontaneous ovulation is preceded by a surge in both follicle stimulating hormone (FSH) and LH, which is thought to induce final oocyte maturation. In the conventional controlled ovarian stimulation (COS) regimen, final follicular maturation is triggered by a single bolus of human chorionic gonadotropin (hCG) which acts as a surrogate to the naturally occurring LH surge to induce luteinization of the granulosa cells, resumption of meiosis and final oocyte maturation [3]. As with other aspects of the COS regimen, triggering of final follicular maturation has become the subject of research interest during the last decade, in an attempt to further improve IVF success rates [4]. It has been demonstrated that ovulation can also be triggered by GnRH agonist, which acts to stimulate the release of endogenous hormones (mainly FSH and LH) required for the final follicular maturation [5]. Subsequently, many studies have compared the effects of hCG trigger versus GnRH agonist trigger in an IVF cycle. Studies examining GnRH agonist single trigger found oocyte and/or embryo quality to be at least comparable leading then to the exploration of the dual trigger, the concomitant administration of GnRH agonist and standard bolus hCG for final follicular maturation [4, 6]. Dual trigger improves oocyte maturation while providing more sustained support for the corpus luteum [7, 8]. Furthermore, the use of dual trigger reduces the required dose of hCG, which is more applicable in women with risk factors for ovarian hyperstimulation syndrome [7, 8].

Several studies have indicated that dual trigger treatment may be associated with increased clinical pregnancy and live birth rates compared with the hCG trigger alone [9–12]. Most of these studies are retrospective cohort studies and limited by potential confounding factors. The results of recent two randomized controlled trials (RCTs) comparing live birth rate (LBR) after dual trigger and single hCG trigger are conflicting [13, 14]. A previous meta-analysis including four randomised trials, showed that dual trigger significantly improved clinical pregnancy rate compared with hCG trigger [15]. However, this meta-analysis did not report on LBR due to the absence of data in all included studies. Additionally, the included studies were of a small sample size and low quality, and therefore the findings warrant further confirmation. Therefore, we aimed to perform a systematic review and meta-analysis to evaluate the effects of dual trigger compared with hCG trigger on IVF outcomes.

## Methods

### Inclusion and exclusion criteria

Only randomized trials that compared the effect of dual trigger with hCG trigger for final oocyte maturation in women undergoing IVF were included. Studies not written in English were excluded. Reviews, conference abstracts, case reports, observational studies, and study protocols were also excluded.

### Literature search

Two authors (KL.H and X.Y) independently searched the database of PubMed, EMBASE, Cochrane Library, Web Of Science up to July 2020. The key search terms included “GnRH agonist”, “dual trigger”, “hCG trigger”, “in vitro fertilization”, “randomized trials”. The detailed search terms and methods could be found in Supplemental Table 1. References from all included studies were also reviewed to identify relevant articles not captured by the electronic searches.

### Study selection

Two authors (KL.H and X.Y) independently scrutinized all of the titles and abstracts according to the predefined inclusion criteria. Full manuscripts of the studies considered for inclusion were then carefully reviewed. Any disagreement towards the study inclusion was resolved by a third author (D.Z).

### Data extraction

Two authors (KL.H and X.Y) independently extracted data from included studies. In cases where we identified a study with multiple publications, the main trial report was used as the reference and additional details were supplemented from other papers.

### Study quality assessment and publication bias

Two reviewers (KL.H and S.W) independently conducted the quality assessment of the included studies. To evaluate the risk of bias, we followed the Cochrane Collaboration’s criteria (Version 2 of the Cochrane risk-of-bias tool for randomized trials (RoB 2)). Funnel plot was used to assess the publication bias for the primary outcome.

### Statistical analysis

The Review Manager version 5 was used to analyze the extracted data. Because studies included in the meta-analysis reported continuous results with different effect size (mean with standard deviation, mean difference with 95% confidence interval (CI), or mean with standard error (SE)), we transformed all of the effect size of continuous outcomes into mean difference with SE according to the Cochrane Handbook for Systematic Reviews of Interventions (version 6.1, 2020. Available from https://training.cochrane.org/handbook/current). The results were combined for meta-analysis using the Mantel/Haenszel model. A fixed-effects model was used where no statistically significant heterogeneity was present (I^2^ < 50%). When substantial heterogeneity was observed (I^2^ > 50%), a random-effect meta-analysis was used. The discontinuous results were shown by risk ratio (RR) with 95% CI. The continuous results were shown by the difference in means with 95% CI. Statistical significance was set at a P level of 0.05. The Grading of Recommendations Assessment, Development, and Evaluation (GRADE) system was used to rate the quality of evidence of studies included in the meta-analysis by two independent reviewers and four levels of evidence were determined (very low, low, moderate, and high) [16].

### Outcomes

The primary outcome was the live birth rate (LBR) per started cycle defined as the total number of participants with at least one baby born after 24 weeks of gestation divided by the total number of started cycles resulting in ovulation trigger. Secondary outcomes included the number of oocytes collected, the number of mature (MII) oocytes collected, the number of fertilised oocytes (2PN), and the number of usable embryos as well as the implantation rate, clinical pregnancy rate, ongoing pregnancy rate, and ovarian hyperstimulation syndrome (OHSS) rate per started cycle. Implantation rate was defined as the number of gestational sacs observed divided by the number of embryos transferred [17, 18]. Clinical pregnancy rate was defined as the number of the cases with a pregnancy diagnosed by ultrasonographic visualization of one or more gestational sacs or definitive clinical signs of pregnancy divided by the total number of started cycles [17, 18]. Ongoing pregnancy rate was defined as the number of cases with at least one live intrauterine fetus after 12 weeks gestation divided by the total number of the started cycles. Ovarian hyperstimulation syndrome was defined as an exaggerated systemic response to ovarian stimulation characterized by a wide spectrum of clinical and laboratory manifestations [17, 18]. The number of usable embryos was defined as the number of embryos at either cleavage or blastocyst stage suitable for transfer and/ or cryopreservation.

## Results

### Characteristics of the included studies

**Fig. 1:**
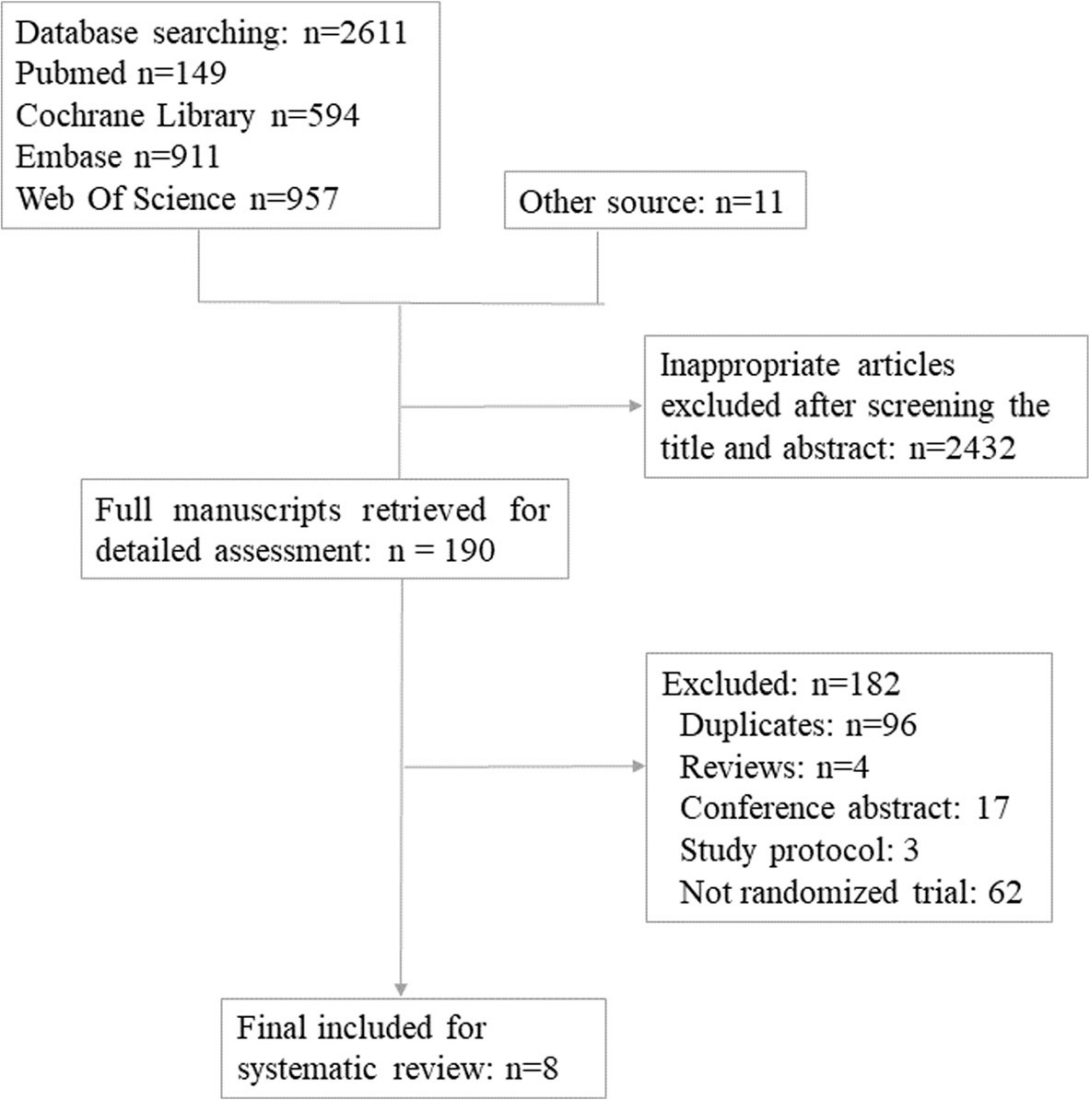
The PRISMA flow diagram of the review process

**Fig. 2:**
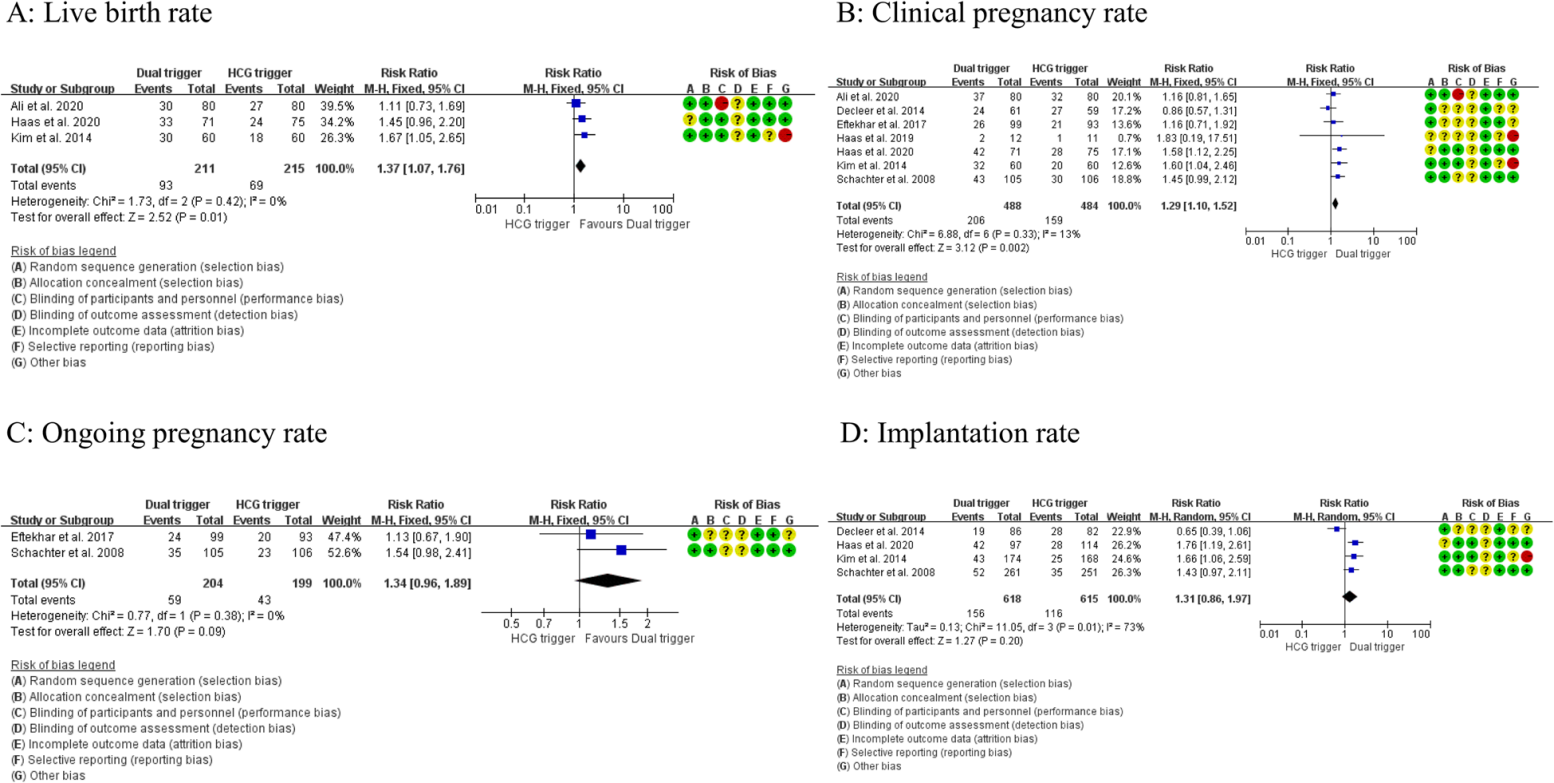
**A**. Meta-analysis of studies reporting the cumulative live birth rate per participant; **B**. Meta-analysis of studies reporting the clinical pregnancy rate per cycle; **C**. Meta-analysis of studies reporting the ongoing pregnancy rate per cycle; **D**. Meta-analysis of studies reporting the implantation rate per cycle

**Fig. 3:**
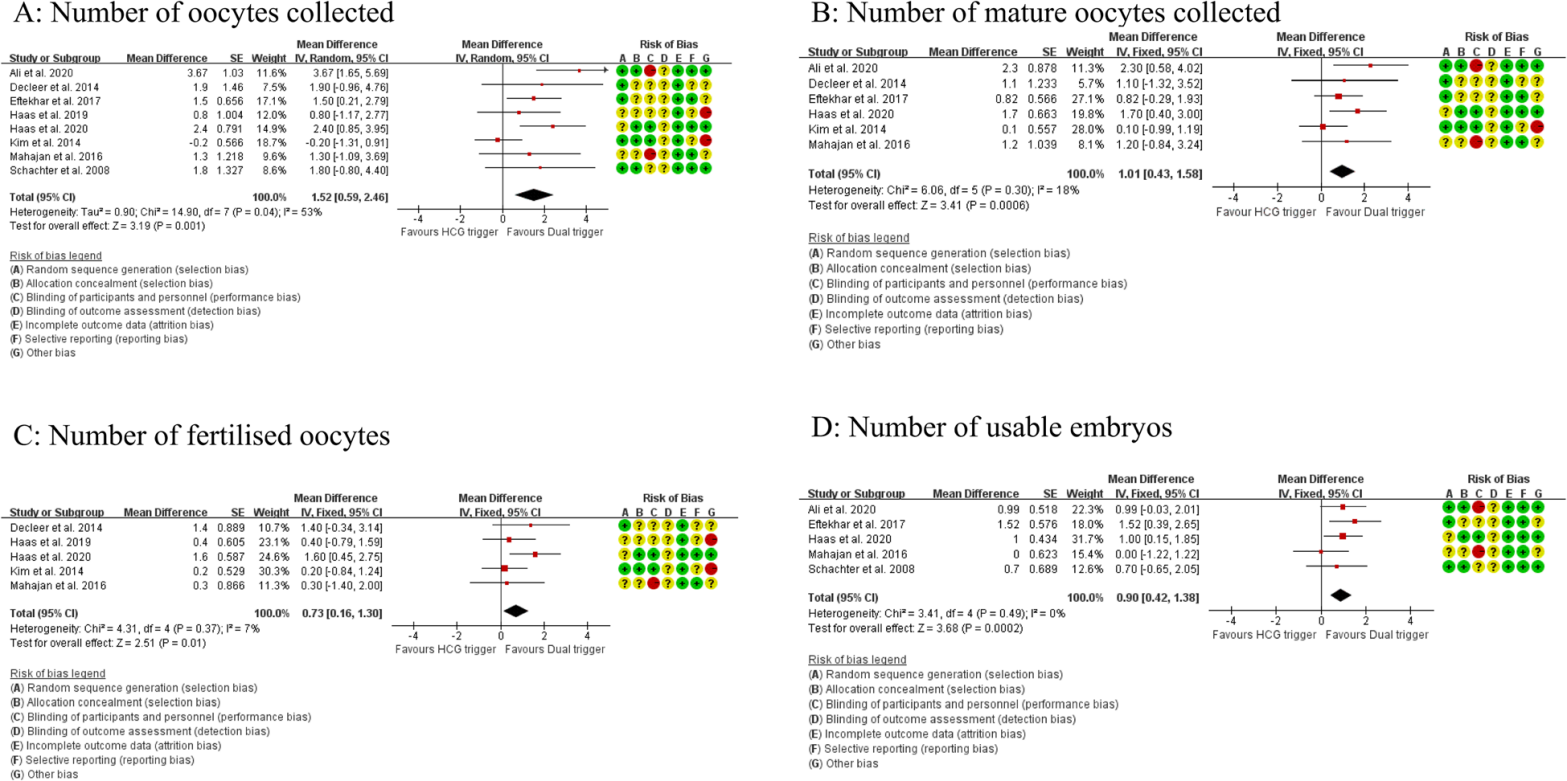
**A**. Meta-analysis of studies reporting the number of oocytes retrieved; **B**. Meta-analysis of studies reporting the number of mature oocytes; **C**. Meta-analysis of studies reporting the number of the fertilized oocytes; **D**. Meta-analysis of studies reporting the number of usable embryos

The PRISMA flow diagram of the review process is presented in Fig. 1. A total of 8 randomized trials were included for analysis and no multiple publications were identified [13, 14, 19–24]. The characteristics of the included studies are presented in Table 1. The included studies were published between 2008 to 2020. A total of 1048 participants were included in the analysis, with 519 in the dual trigger group (intervention group) and 529 in the hCG trigger group (control group). Sample sizes varied from 23 women to 211 women. Participants were randomized on the day of trigger in two of the included studies [14, 24] and the day that the leading follicles reached 17 mm in diameter in one study [20]. The remaining studies did not specify the time of randomization. Most trials enrolled women with an age range from 18 to 42 years and body mass index (BMI) 18.0–30.0 kg/m^2^ [13, 14, 20, 23, 24]. Three trials excluded women with polycystic ovarian syndrome [20, 22, 23]. One trial enrolled women with poor ovarian response defined by Bologna criteria [19]. One trial enrolled women with a moderate ovarian response [20]. The overall participant population included mostly women with expected normal ovarian response based on age, BMI and baseline AFC, FSH and AMH levels, excluding PCOS (*n* = 3) or patients with established or increased risk for a high ovarian response. The exception was a single study performed in poor responders with a small sample size [19]. Therefore, a sensitivity analysis was conducted by excluding this study. The detailed inclusion and exclusion criteria in each study are summarized in Table 1. The GnRH antagonist protocol for pituitary down-regulation was used in all of the included studies. In the intervention group, the dose of GnRH agonist varied from 0.1 mg to 1 mg and the dose of hCG was the same with the control group in all studies except Mahajan (2016), which used 5000 IU hCG in the intervention group and 10,000 IU in the control group. One study was a double-blind design with the placebo and 10,000 IU hCG used as control [14]. Three studies reported live birth per cycle [13, 14, 22]. The secondary outcomes reported in each study are summarized in Table 1. All studies included for meta-analysis were randomised trials. Assessment for risk of bias is displayed in Figs. 2 and 3. Most studies were at high risk of bias and only two studies were of good quality [25, 26]. The funnel plot showed no significant publication bias for the included studies (Supplemental Figure 1). Risk of bias summary and each risk of bias item are presented as percentages across all included studies and are displayed in Supplemental Figure 2 and Supplemental Figure 3, respectively. The outcomes reported in each study and definitions for the outcomes were summarized in Table 2. Notably, Haas et al. (2019) and Decleer et al. (2014) reported the outcome of ongoing pregnancy but in fact it was clinical pregnancy. In our meta-analysis, the data was extracted as the clinical pregnancy (Fig. 2).

**Table 1:** Characteristics of inculded studies

Author (year)	Conflict of interest	Ethical approval	Time of randomization	Time frame	Inclusion criteria	Exclusion criteria	Sample size	Intervention	Comparison
Haas et al. (2020) [14]	None	Yes	Morning of the trigger day	2016–2018	Age 18–41 y; BMI 18–35 kg/m2; AMH > 1 ng/ml; AFC 6–20; FSH < 20 IU/l; first three IVF cycle	E2 levels > 15,000 pmol/l; moderate–severe endometriosis	146	hCG 10,000 IU and GnRH agonist 0.5 mg	hCG 10,000 IU and placebo
Mahajan et al. (2016) [21]	None	Yes	NR	NR	Aged 24–43; AMH < 4 ng/ml; AFCs/ovary < 12	None	76	hCG 5000 IU and GnRH agonist 1 mg	hCG 10,000 IU
Ali et al. (2020) [13]	None	Yes	NR	2016–2018	First ICSI cycle; aged less than 40 y; BMI 18–30 kg/m2; AMH > 1 ng/ml; normal, mild or moderate male factor infertility	Azoospermic males	160	250 IU of recombinant HCG and GnRH agonist 1 mg	250 IU of recombinant HCG
Schachter et al. (2008) [24]	NR	Yes	Onset of intervention	NR	Failed at least one IVF-ET cycle on GnRH agonist long protocol; hysterosalpingogram or hysteroscopy history; BMI 18–30 kg/m2;	Lack of oocytes aspirated in previous cycles;	211	hCG 5000 IU and GnRH agonist 0.2 mg	hCG 5000 IU
Haas et al. (2019) [19]	None	Yes	NR	2015–2017	Poor responders defined with the Bologna criteria.	None	23	hCG 6500 IU and GnRH agonist (dose not reported)	hCG 6.500 IU
Eftekhar et al. (2017) [20]	None	Yes	Leading follicles reached 17 mm in diameter	2014–2015	Aged less than 42 y; BMI 18–30 kg/m2; moderate ovarian response	Endocrine disorders; PCOS; UA; RIF; azoospermia; day-3 FSH ≥ 10 IU/L or AMH ≤1.0 ng/mL	192	hCG 6500 IU and GnRH agonist 0.2 mg	hCG 6.500 IU
Kim et al. (2014) [22]	NR	Yes	NR	NR	Women with regular ovulatory cycles	PCOS, metabolic disorders	120	250 μg rhCG and GnRH agonist 0.1 mg	250 μg rhCG and placebo
Decleer et al. (2014) [23]	NR	Yes	NR	2011–2013	Tubal or male infertility, BMI < 32, age ≤ 38 y, frst, second and third IVF cycle.	Azospermia, UA, PCOS, endocrine disorders, endometriosis	120	hCG 5000 IU and GnRH agonist 0.2 mg	hCG 5000 IU

*Abbreviations*: *NR* not reported, *NS* no special, *ET* embryo transfer, *IVF* in vitro fertilization, *ICSI* intracytoplasmic sperm injection, *2PN* two pronuclei, *OHSS* ovarian hyperstimulation syndrome, *PCOS* polycystic ovarian syndrome, *BMI* body mass index, *PGT* preimplantation genetic screening, *RIF* repeated implantation failure, *UA* uterine anomalies, *AMH* anti-Mullerian Hormone

**Table 2:** Outcomes with definitions of the inculded studies

Author (year)	Outcomes reported	Primary outcome	Outcomes not reported	Outcomes definition in each study
Haas et al. (2020) [14]	Number of mature oocytes; total number of oocytes, 2PN zygotes, embryo number, clinical pregnancy rate, implantation rate, live birth rate	Number of mature oocytes	Ongoing pregnancy rate	Number of mature oocytes: number of MII oocytes. Other outcomes were not defined.
Mahajan et al. (2016) [21]	Number of mature oocytes; total number of oocytes, 2PN zygotes; usable embryos	Number of mature oocytes; usable embryos	Ongoing pregnancy rate, clinical pregnancy rate, implantation rate, live birth rate	Usable embryos: embryos good for transfer and cryopreservation. Other outcomes were not defined.
Ali et al. (2020) [13]	Number of mature oocytes; total number of oocytes; number of Grade 1 embryos; fertilization rate (without the exact fertilizaton number); implantation rate (without the exact sac number and transferred embryo number); clinical pregnancy rate; live birth rate	Number of mature oocytes	Ongoing pregnancy rate	Number of mature oocytes: number of MII oocytes; fertilization rate: the number of the fertilized oocytes divided by the total number of the retrieved oocytes per 100; implantation rate: total number of the sacs divided by the total number of the transferred embryos per 100; clinical pregnancy rate: the number of the cases with at least one sac in or out the uterus divided by the cycles initiated per 100; live birth rate: total number of the cases with at least one baby born after 24 weeks of gestation divided by the total number of the cycles initiated per 100.
Schachter et al. (2008) [24]	Total number of oocytes; usable embryos; ongoing pregnancy rate; clinical pregnancy rate; implantation rate	Not mentioned	Number of mature oocytes; 2PN zygotes; live birth rate	Implantation rate: number of gestational sacs per embryo transferred. Other outcomes were not defined.
Haas et al. (2019) [19]	Total number of oocytes; 2PN zygotes; ongoing pregnancy rate	Total number of oocytes, top-quality embryo	Number of mature oocytes, clinical pregnancy rate, implantation rate, live birth rate, usable embryos	Ongoing pregnancy: visualization of a gestational sac and fetal cardiac activity on transvaginal ultrasound. Other outcomes were not defined.
Eftekhar et al. (2017) [20]	Number of mature oocytes; total number of oocytes, usable embryos; implantation number, clinical and ongoing pregnancy	Clinical pregnancy	2PN zygotes, live birth rate	No definition of outcomes
Kim et al. (2014) [22]	Number of mature oocytes; total number of oocytes, 2PN zygotes, top-quality embryo, clinical pregnancy, implantation rate, live birth rate, severe OHSS	Not mentioned	Ongoing pregnancy rate, usable embryos	Clinical pregnancy: increased serum β-hCG concentration. Other outcomes were not defined.
Decleer et al. (2014) [23]	Number of mature oocytes; total number of oocytes, 2PN zygotes; Implantation rate; ongoing pregnancy rate	Number of mature oocytes	Clinical pregnancy rate, live birth rate, usable embryos	Number of mature oocytes: number of MII oocytes. Implantation rate: mean number of foetal sacs per embryo transferred. Ongoing pregnancy: confirmed by ultrasound 23 days after embryo transfer. Other outcomes were not defined.

*Abbreviations*: *2PN* two pronuclei, *OHSS* ovarian hyperstimulation syndrome

### Primary outcome

#### LBR per started cycle

Three studies compared the live birth rate per cycle between the dual trigger group and the hCG trigger group (Fig. 2a). Meta-analysis suggested that the dual trigger treatment was associated with a significantly higher live birth rate per cycle than the hCG trigger (RR = 1.37 [1.07, 1.76], I^2^ = 0%, moderate evidence).

### Secondary outcome

#### Clinical pregnancy rate per started cycle

Seven studies reported clinical pregnancy rate (Fig. 2b). Meta-analysis suggested that dual trigger treatment was associated with a significant increase in clinical pregnancy rate compared with hCG trigger treatment (RR = 1.29 [1.10, 1.52], I^2^ = 13%, low evidence). A sensitivity analysis, excluding the study done in poor responders, showed dual trigger treatment significantly increased the clinical pregnancy rate (RR = 1.29 [1.10, 1.52], I^2^ = 26%, low evidence) [19].

#### Ongoing pregnancy rate per cycle

Two studies reported the ongoing pregnancy rate (Fig. 2c). Meta-analysis suggested that dual trigger was associated with a non-significant increase in ongoing pregnancy rate (RR = 1.34 [0.96, 1.89], I^2^ = 0%, low evidence).

#### Implantation rate per cycle

Three studies reported implantation rate (Fig. 2d). Meta-analysis suggested that dual trigger treatment was associated with a non-significant increase in implantation rate (RR = 1.31 [0.86, 1.97], I^2^ = 73%, low evidence). A sensitivity analysis, excluding the study with low quality and induced the significant heterogeneity of meta-analysis showed dual trigger treatment significantly increased the implantation rate (RR = 1.61 [1.27, 2.03], I^2^ = 0%, moderate evidence) [23].

### The mean difference in the number of oocytes retrieved

The data regarding the number of oocytes collected was available to be extracted and synthesized in all of the included studies (Fig. 3a). Meta-analysis suggested that dual trigger treatment was associated with a significant increase in the number of oocytes collected (MD = 1.52 [0.59, 2.46], I^2^ = 53%, low evidence). A sensitivity analysis, excluding the study done in poor responders showed dual trigger treatment confirmed this finding (MD = 1.64 [0.58, 2.70], I^2^ = 59%, low evidence) [19].

### The mean difference in maturate oocytes collected

Data regarding the number of maturated oocytes collected was available to be extracted and synthesized in six studies (Fig. 3b). Meta-analysis suggested that dual trigger treatment was associated with a significant increase in the number of maturate oocytes collected (MD = 1.01 [0.43, 1.58], I^2^ = 18%, low evidence).

### The mean difference in the number of fertilized oocytes

Data comparing the number of fertilized oocytes was available to be extracted and synthesized in five studies (Fig. 3c). Meta-analysis suggested that dual trigger treatment was associated with a significant increase in the number of fertilized oocytes (MD = 0.73 [0.16, 1.30], I^2^ = 7%, low evidence). A sensitivity analysis, excluding the study done in poor responders, showed similar association (MD = 0.85 [0.18, 1.48], I^2^ = 24%, low evidence) [19].

### The mean difference in the number of usable embryos

Data regarding the comparison of the number of usable embryos was available to be extracted and synthesized in five studies (Fig. 3d). Meta-analysis suggested that dual trigger treatment was associated with a significant increase in the number of usable embryos (MD = 0.90 [0.42, 1.38], I^2^ = 0%, low evidence).

### OHSS per started cycle

Three studies reported on OHSS rate in the participants. Dual trigger treatment was not associated with an increase in OHSS (RR = 1.00 [0.14, 7.34]) (Supplemental Figure 4) however none of the three studies had a clear definition of OHSS. There were no cases of OHSS in two of the included studies [14, 23]. Another study reported four OHSS cases, with two cases in the dual trigger group and two in the hCG group [22].

## Discussion

This systematic review included eight randomized control studies for quantitative analysis. We found that dual trigger treatment was associated with a significantly higher LBR per cycle than the single hCG trigger (moderate evidence).

It is proposed that GnRH agonist administration induces an FSH surge in addition to the LH surge which better mimics normal physiology. This promotes the dissociation of the oocyte from the follicular wall and the formation of LH receptors in luteinizing granulosa cells as well as keeping gap junctions open between the oocyte and cumulus cells and promoting oocyte maturation and cumulus expansion [27]. Previous studies demonstrate that both FSH and LH hormones are significantly elevated after GnRH agonist treatment [6, 24, 28]. Furthermore, in animal studies, increasing the incubation concentrations of GnRH agonist significantly enhances preimplantation embryonic development, indicating a direct GnRH–GnRH receptor activation on the embryo [29]. GnRH agonist administration may also facilitate embryo implantation by modulating corpus luteum function, or in a direct effect on the endometrium [30].

Studies regarding the possible benefits of dual triggering are conflicting. Emerging evidence suggests that the total number of retrieved oocytes, the number of mature oocytes, and usable embryos are significantly increased after triggering with GnRH agonist and hCG compared with the conventional triggering with hCG alone [11, 31]. Furthermore, dual trigger treatment was associated with increased implantation and clinical pregnancy rates [9–12, 31]. However, most of these studies are retrospective in design and thereby limited by potential confounding factors. A previous meta-analysis of four RCTs found no significant difference between the two trigger treatment groups in terms of the number of total oocytes retrieved, the number of mature (MII) oocytes retrieved, the number of fertilized oocytes or the implantation rate. However, the number of good-quality embryos and the ongoing pregnancy rate was significantly increased in the dual trigger group [15]. This meta-analysis study was limited by the small number of included trials and the small sample sizes of these studies [15]. Additionally, none of the included RCTs reported on live birth rate [15]. Therefore the finding of no difference may be explained by the small sample size because our study also concluded that there was no significant increase in implantation rate (four included RCTs) and ongoing pregnancy rate (two RCTs) after the dual trigger. When we excluded the low-quality study for sensitivity analysis, the dual trigger was demonstrably associated with a significant increase in implantation rate. Although ongoing pregnancy rate was not significantly increased in the dual trigger group, the risk ratio was quite similar to live birth rate (1.34 versus 1.31), indicating that the small sample size may be responsible for the finding of no difference. These results highlight that future trials should better be designed well and report all available outcomes. Our study also suggests that the number of oocytes retrieved, mature MII oocytes retrieved, fertilised oocytes, and usable embryos are significantly increased after dual trigger treatment. The larger number of included studies and the larger sample size in our meta-analysis may explain this finding. Although our study shows that dual trigger treatment was associated with a significant increase in LBR, only three studies report this outcome. Future studies should report this important outcome.

It should be noted that one trial included in our meta-analysis enrolled patients with a poor ovarian response [19], while other trials enrolled women with a moderate response or used ovarian reserve markers in the inclusion criteria. The heterogeneity of the participants makes it difficult to conduct subgroup analysis in women with different ovarian responses. Only 4 OHSS cases were reported, with two cases in the dual trigger and another two in the hCG trigger group. The results indicate that dual trigger is unlikely to increase the OHSS rate compared to hCG trigger treatment. Previous retrospective studies indicate that dual trigger can reduce the OHSS rate in predicted high responders [32, 33]. Because most trials included in our study enroll women with a moderate response, whether the dual trigger is more applicable in high or moderate or poor responders requires further confirmation [10, 32, 33]. Additionally, no consensus has been reached towards the best dose of GnRH agonist and hCG in the dual trigger treatment. The possibility to use a low-dose of hCG in the dual trigger treatment may be limited by the lack of low-dose hCG formulations.

The strength of this study is that only randomised trials were included in the meta-analysis. Additionally, the evidence towards the comparison of live birth rate between the dual trigger and the hCG trigger is moderate. The main limitation of this study is the low quality of most studies included in the meta-analysis and the risk of bias associated with poor reporting of methods in the included studies. More robust evidence should be provided by trials with a larger sample size and good design.

In conclusion, this systematic review suggests that dual trigger treatment is associated with a significant increase in clinical pregnancy and LBR in IVF cycles and that the effect is potentially mediated by an increase in the quantity and quality of oocytes and embryos.

## Supplementary Information

**Additional file 1: Supplemental figure 1.** Funnel plot of the included studies.

**Additional file 2: Supplemental figure 2.** Risk of bias summary: review authors' judgments about each risk of bias item for each included study.

**Additional file 3: Supplemental figure 3.** Risk of bias graph: review authors' judgments about each risk of bias item presented as percentages across all included studies.

**Additional file 4: Supplemental figure 4.** Meta-analysis of studies reporting the number of the OHSS rate.

**Additional file 5: Supplementary table 1**.

## Data Availability

All data generated or analysed during this study are included in this published article.

## Abbreviations

hCG: Human chorionic gonadotropin
LH: Luteinizing hormone
GnRH: Gonadotropin-releasing hormone
IVF: In vitro fertilization
FSH: Follicle stimulating hormone
COS: Controlled ovarian stimulation
RR: Risk ratio
MD: Mean difference
LBR: Live birth rate
RCT: Randomsed controlled trial
CI: Confidence interval
SE: Standard error
OHSS: Ovarian hyperstimulation syndrome
BMI: Body mass index

## References

[1] Thoma ME, McLain AC, Louis JF, King RB, Trumble AC, Sundaram R (2013). Prevalence of infertility in the United States as estimated by the current duration approach and a traditional constructed approach. Fertil Steril.

[2] Zhou Z, Zheng D, Wu H, Li R, Xu S, Kang Y, Cao Y, Chen X, Zhu Y, Xu S, Chen ZJ, Mol BW, Qiao J (2018). Epidemiology of infertility in China: a population-based study. BJOG.

[3] Ludwig M, Doody KJ, Doody KM (2003). Use of recombinant human chorionic gonadotropin in ovulation induction. Fertil Steril.

[4] Orvieto R (2015). Triggering final follicular maturation--hCG, GnRH-agonist or both, when and to whom?. J Ovarian Res.

[5] Gonen Y, Balakier H, Powell W, Casper RF (1990). Use of gonadotropin-releasing hormone agonist to trigger follicular maturation for in vitro fertilization. J Clin Endocrinol Metab.

[6] Humaidan P, Bredkjaer HE, Bungum L, Bungum M, Grøndahl ML, Westergaard L (2005). GnRH agonist (buserelin) or hCG for ovulation induction in GnRH antagonist IVF/ICSI cycles: a prospective randomized study. Hum Reprod.

[7] Shapiro BS, Daneshmand ST, Garner FC, Aguirre M, Thomas S (2008). Gonadotropin-releasing hormone agonist combined with a reduced dose of human chorionic gonadotropin for final oocyte maturation in fresh autologous cycles of in vitro fertilization. Fertil Steril.

[8] Humaidan P, Papanikolaou EG, Tarlatzis BC (2009). GnRHa to trigger final oocyte maturation: a time to reconsider. Hum Reprod.

[9] Lin MH, Wu FS, Hwu YM, Lee RK, Li RS, Li SH (2019). Dual trigger with gonadotropin releasing hormone agonist and human chorionic gonadotropin significantly improves live birth rate for women with diminished ovarian reserve. Reprod Biol Endocrinol.

[10] Lin MH, Wu FS, Lee RK, Li SH, Lin SY, Hwu YM (2013). Dual trigger with combination of gonadotropin-releasing hormone agonist and human chorionic gonadotropin significantly improves the live-birth rate for normal responders in GnRH-antagonist cycles. Fertil Steril.

[11] Chern CU, Li JY, Tsui KH, Wang PH, Wen ZH, Lin LT (2020). Dual-trigger improves the outcomes of in vitro fertilization cycles in older patients with diminished ovarian reserve: a retrospective cohort study. PLoS One.

[12] Griffin D, Benadiva C, Kummer N, Budinetz T, Nulsen J, Engmann L (2012). Dual trigger of oocyte maturation with gonadotropin-releasing hormone agonist and low-dose human chorionic gonadotropin to optimize live birth rates in high responders. Fertil Steril.

[13] Ali SS, Elsenosy E, Sayed GH, Farghaly TA, Youssef AA, Badran E, Abbas AM, Abdelaleem AA (2020). Dual trigger using recombinant HCG and gonadotropin-releasing hormone agonist improve oocyte maturity and embryo grading for normal responders in GnRH antagonist cycles: randomized controlled trial. J Gynecol Obstet Hum Reprod.

[14] Haas J, Bassil R, Samara N, Zilberberg E, Mehta C, Orvieto R, Casper RF (2020). GnRH agonist and hCG (dual trigger) versus hCG trigger for final follicular maturation: a double-blinded, randomized controlled study. Hum Reprod.

[15] Chen CH, Tzeng CR, Wang PH, Liu WM, Chang HY, Chen HH, Chen CH (2018). Dual triggering with GnRH agonist plus hCG versus triggering with hCG alone for IVF/ICSI outcome in GnRH antagonist cycles: a systematic review and meta-analysis. Arch Gynecol Obstet.

[16] Guyatt G, Oxman AD, Akl EA, Kunz R, Vist G, Brozek J, Norris S, Falck-Ytter Y, Glasziou P, DeBeer H, Jaeschke R, Rind D, Meerpohl J, Dahm P, Schünemann HJ (2011). GRADE guidelines: 1. Introduction—GRADE evidence profiles and summary of findings tables. J Clin Epidemiol.

[17] Zegers-Hochschild F, Adamson GD, Dyer S, Racowsky C, de Mouzon J, Sokol R, Rienzi L, Sunde A, Schmidt L, Cooke ID, Simpson JL, van der Poel S (2017). The international glossary on infertility and fertility care, 2017. Hum Reprod.

[18] Zegers-Hochschild F, Adamson GD, Dyer S, Racowsky C, de Mouzon J, Sokol R, Rienzi L, Sunde A, Schmidt L, Cooke ID, Simpson JL, van der Poel S (2017). The international glossary on infertility and fertility care, 2017. Fertil Steril.

[19] Haas J, Zilberberg E, Nahum R, Mor Sason A, Hourvitz A, Gat I, Orvieto R (2019). Does double trigger (GnRH-agonist + hCG) improve outcome in poor responders undergoing IVF-ET cycle? A pilot study. Gynecol Endocrinol.

[20] Eftekhar M, Mojtahedi MF, Miraj S, Omid M (2017). Final follicular maturation by administration of GnRH agonist plus HCG versus HCG in normal responders in ART cycles: An RCT. Int J Reprod Biomed.

[21] Mahajan N, Sharma S, Arora PR, Gupta S, Rani K, Naidu P (2016). Evaluation of dual trigger with gonadotropin-releasing hormone agonist and human chorionic gonadotropin in improving oocyte maturity rates: a prospective randomized study. J Hum Reprod Sci.

[22] Kim CH, Ahn JW, You RM, Kim SH, Chae HD, Kang BM (2014). Combined administration of gonadotropin-releasing hormone agonist with human chorionic gonadotropin for final oocyte maturation in GnRH antagonist cycles for in vitro fertilization. J Reprod Med.

[23] Decleer W, Osmanagaoglu K, Seynhave B, Kolibianakis S, Tarlatzis B, Devroey P (2014). Comparison of hCG triggering versus hCG in combination with a GnRH agonist: a prospective randomized controlled trial. Facts Views Vision ObGyn.

[24] Schachter M, Friedler S, Ron-El R, Zimmerman AL, Strassburger D, Bern O (2008). Can pregnancy rate be improved in gonadotropin-releasing hormone (GnRH) antagonist cycles by administering GnRH agonist before oocyte retrieval? A prospective, randomized study. Fertil Steril.

[25] Fernando S, Wallace EM, Vollenhoven B, Lolatgis N, Hope N, Wong M, Lawrence M, Lawrence A, Russell C, Leong K, Thomas P, Rombauts L (2018). Melatonin in assisted reproductive technology: a pilot double-blind randomized placebo-controlled clinical trial. Front Endocrinol.

[26] Mokhtari F, Akbari Asbagh F, Azmoodeh O, Bakhtiyari M, Almasi-Hashiani A (2019). Effects of melatonin administration on chemical pregnancy rates of polycystic ovary syndrome patients undergoing intrauterine insemination: a randomized clinical trial. Int J Fertil Steril.

[27] Mizrachi Y, Horowitz E, Farhi J, Raziel A, Weissman A (2020). Ovarian stimulation for freeze-all IVF cycles: a systematic review. Hum Reprod Update.

[28] Fauser BC, de Jong D, Olivennes F, Wramsby H, Tay C, Itskovitz-Eldor J, van Hooren HG (2002). Endocrine profiles after triggering of final oocyte maturation with GnRH agonist after cotreatment with the GnRH antagonist ganirelix during ovarian hyperstimulation for in vitro fertilization. J Clin Endocrinol Metab.

[29] Raga F, Casañ EM, Kruessel J, Wen Y, Bonilla-Musoles F, Polan ML (1999). The role of gonadotropin-releasing hormone in murine preimplantation embryonic development. Endocrinology.

[30] Tesarik J, Hazout A, Mendoza-Tesarik R, Mendoza N, Mendoza C (2006). Beneficial effect of luteal-phase GnRH agonist administration on embryo implantation after ICSI in both GnRH agonist- and antagonist-treated ovarian stimulation cycles. Hum Reprod.

[31] Griffin D, Feinn R, Engmann L, Nulsen J, Budinetz T, Benadiva C (2014). Dual trigger with gonadotropin-releasing hormone agonist and standard dose human chorionic gonadotropin to improve oocyte maturity rates. Fertil Steril.

[32] Engmann L, DiLuigi A, Schmidt D, Nulsen J, Maier D, Benadiva C (2008). The use of gonadotropin-releasing hormone (GnRH) agonist to induce oocyte maturation after cotreatment with GnRH antagonist in high-risk patients undergoing in vitro fertilization prevents the risk of ovarian hyperstimulation syndrome: a prospective randomized controlled study. Fertil Steril.

[33] Li S, Zhou D, Yin T, Xu W, Xie Q, Cheng D, Yang J (2018). Dual trigger of triptorelin and HCG optimizes clinical outcome for high ovarian responder in GnRH-antagonist protocols. Oncotarget.

