# Supplementary figures and images for: GnRH agonist and hCG (dual trigger) versus hCG trigger for follicular maturation: a systematic review and meta-analysis of randomized trials
Source: Reprod Biol Endocrinol. 2021 Jun 1;19:78. doi: 10.1186/s12958-021-00766-5 (PMC8167939; doi:10.1186/s12958-021-00766-5)

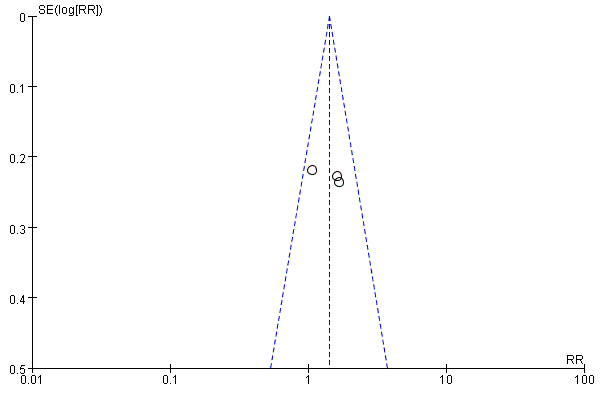

Supplement: Supplementary file 1 — Additional file 1: Supplemental figure 1. Funnel plot of the included studies. [file 12958_2021_766_MOESM1_ESM.png]

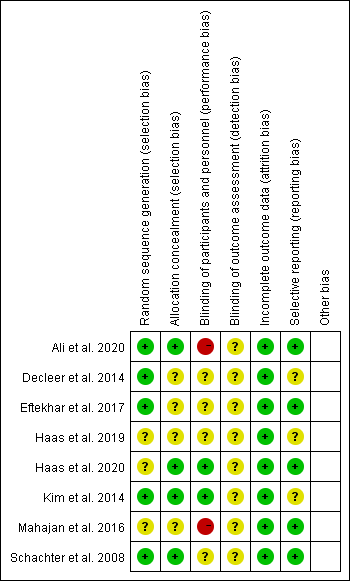

Supplement: Supplementary file 2 — Additional file 2: Supplemental figure 2. Risk of bias summary: review authors' judgments about each risk of bias item for each included study. [file 12958_2021_766_MOESM2_ESM.png]

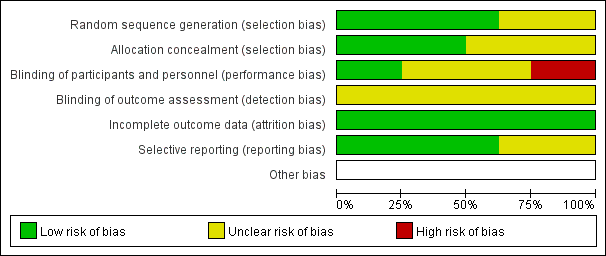

Supplement: Supplementary file 3 — Additional file 3: Supplemental figure 3. Risk of bias graph: review authors' judgments about each risk of bias item presented as percentages across all included studies. [file 12958_2021_766_MOESM3_ESM.png]

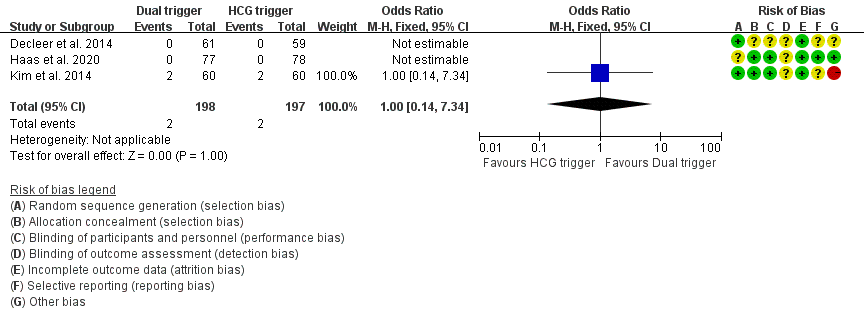

Supplement: Supplementary file 4 — Additional file 4: Supplemental figure 4. Meta-analysis of studies reporting the number of the OHSS rate. [file 12958_2021_766_MOESM4_ESM.png]
