# Supplementary material for: GnRH agonist and hCG (dual trigger) versus hCG trigger for follicular maturation: a systematic review and meta-analysis of randomized trials
Source: Reprod Biol Endocrinol. 2021 Jun 1;19:78. doi: 10.1186/s12958-021-00766-5 (PMC8167939; doi:10.1186/s12958-021-00766-5)
Supplement: Supplementary file 5 — Additional file 5: Supplementary table 1. [file 12958_2021_766_MOESM5_ESM.docx]

Pubmed

| #1 | (((((((((((((Fertilization in Vitro[MeSH Terms]) OR (In Vitro Fertilization[Title/Abstract])) OR (In Vitro Fertilizations[Title/Abstract])) OR (Test-Tube Fertilization[Title/Abstract])) OR (Fertilization, Test-Tube[Title/Abstract])) OR (Fertilizations, Test-Tube[Title/Abstract])) OR (Test Tube Fertilization[Title/Abstract])) OR (test tube fertilization[Title/Abstract])) OR (fertilization in vitro[Title/Abstract])) OR (Test-Tube Babies[Title/Abstract])) OR (Babies, Test-Tube[Title/Abstract])) OR (Baby, Test-Tube[Title/Abstract])) OR (Test Tube Babies[Title/Abstract])) OR (Test-Tube Baby[Title/Abstract]) |
| --- | --- |
| #2 | ((((((((Sperm Injections, Intracytoplasmic[MeSH Terms]) OR (Sperm Injections, Intracytoplasmic[Title/Abstract])) OR (Injection, Intracytoplasmic Sperm[Title/Abstract])) OR (Injections, Intracytoplasmic Sperm[Title/Abstract])) OR (Intracytoplasmic Sperm Injection[Title/Abstract])) OR (Sperm Injection, Intracytoplasmic[Title/Abstract])) OR (Intracytoplasmic Sperm Injections[Title/Abstract])) OR (ICSI[Title/Abstract])) OR (Injections, Sperm, Intracytoplasmic[Title/Abstract]) |
| #3 | (((((((((((((((((((((((((((((((((((((((((((((((((((((((((((((((((((((((((((((((((((((((((((((((((((((((((Gonadotropin-Releasing Hormone[MeSH Terms]) OR (Buserelin[MeSH Terms])) OR (Goserelin[MeSH Terms])) OR (Leuprolide[MeSH Terms])) OR (Nafarelin[MeSH Terms])) OR (Triptorelin Pamoate[MeSH Terms])) OR (Gonadotropin-Releasing Hormone[Title/Abstract])) OR (Buserelin[Title/Abstract])) OR (Goserelin[Title/Abstract])) OR (Leuprolide[Title/Abstract])) OR (Nafarelin[Title/Abstract])) OR (Triptorelin Pamoate[Title/Abstract])) OR (ganirelix[Title/Abstract])) OR (teverelix[Title/Abstract])) OR (Gonadotropin Releasing Hormone[Title/Abstract])) OR (Gn-RH[Title/Abstract])) OR (Gonadoliberin[Title/Abstract])) OR (LHFSH Releasing Hormone[Title/Abstract])) OR (Releasing Hormone, LHFSH[Title/Abstract])) OR (LH-RH[Title/Abstract])) OR (LFRH[Title/Abstract])) OR (LH-Releasing Hormone[Title/Abstract])) OR (LH Releasing Hormone[Title/Abstract])) OR (LH-FSH Releasing Hormone[Title/Abstract])) OR (LH FSH Releasing Hormone[Title/Abstract])) OR (LHFSHRH[Title/Abstract])) OR (LHRH[Title/Abstract])) OR (Luliberin[Title/Abstract])) OR (Gonadorelin[Title/Abstract])) OR (Luteinizing Hormone-Releasing Hormone[Title/Abstract])) OR (Luteinizing Hormone Releasing Hormone[Title/Abstract])) OR (FSH-Releasing Hormone[Title/Abstract])) OR (FSH Releasing Hormone[Title/Abstract])) OR (GnRH[Title/Abstract])) OR (Factrel[Title/Abstract])) OR (Cystorelin[Title/Abstract])) OR (Gonadorelin Hydrochloride[Title/Abstract])) OR (Kryptocur[Title/Abstract])) OR (Dirigestran[Title/Abstract])) OR (Gonadorelin Acetate[Title/Abstract])) OR (Buserelin Acetate[Title/Abstract])) OR (Acetate, Buserelin[Title/Abstract])) OR (Suprefact[Title/Abstract])) OR (Profact[Title/Abstract])) OR (Receptal[Title/Abstract])) OR (Tiloryth[Title/Abstract])) OR (Suprecur[Title/Abstract])) OR (Bigonist[Title/Abstract])) OR (HOE-766[Title/Abstract])) OR (HOE 766[Title/Abstract])) OR (HOE766[Title/Abstract])) OR (ICI-118630[Title/Abstract])) OR (ICI 118630[Title/Abstract])) OR (ICI118630[Title/Abstract])) OR (Zoladex[Title/Abstract])) OR (Goserelin Acetate[Title/Abstract])) OR (Acetate, Goserelin[Title/Abstract])) OR (Leuprorelin[Title/Abstract])) OR (Enantone[Title/Abstract])) OR (Leuprolide Acetate[Title/Abstract])) OR (Acetate, Leuprolide[Title/Abstract])) OR (Leuprolide Monoacetate[Title/Abstract])) OR (Monoacetate, Leuprolide[Title/Abstract])) OR (Leuprolide, (L-Leu)-Isomer[Title/Abstract])) OR (Lupron[Title/Abstract])) OR (TAP-144[Title/Abstract])) OR (TAP 144[Title/Abstract])) OR (TAP144[Title/Abstract])) OR (A-43818[Title/Abstract])) OR (A 43818[Title/Abstract])) OR (A43818[Title/Abstract])) OR (Leuprolide, (DL-Leu)-Isomer[Title/Abstract])) OR (Synarel[Title/Abstract])) OR (RS-94991-298[Title/Abstract])) OR (RS 94991 298[Title/Abstract])) OR (RS94991298[Title/Abstract])) OR (Nafarelin Acetate[Title/Abstract])) OR (Nafarelin Acetate, Hydrate[Title/Abstract])) OR (Nafarelin Monoacetate[Title/Abstract])) OR (Pamoate, Triptorelin[Title/Abstract])) OR (Triptorelin Embonate[Title/Abstract])) OR (Embonate, Triptorelin[Title/Abstract])) OR (CL-118532[Title/Abstract])) OR (CL 118532[Title/Abstract])) OR (CL118532[Title/Abstract])) OR (Trelstar[Title/Abstract])) OR (Triptorelin[Title/Abstract])) OR (LHRH, Trp(6)-[Title/Abstract])) OR (GnRH, Trp(6)-[Title/Abstract])) OR (LHRH, Tryptophyl(6)-[Title/Abstract])) OR (D-Trp-6-LH-RH[Title/Abstract])) OR (6-D-Tryptophan-Luteinizing Hormone-Releasing Factor (Pig)[Title/Abstract])) OR (Wy-42462[Title/Abstract])) OR (Wy 42462[Title/Abstract])) OR (Wy42462[Title/Abstract])) OR (AY-25650[Title/Abstract])) OR (AY 25650[Title/Abstract])) OR (AY25650[Title/Abstract])) OR (Decapeptyl[Title/Abstract])) OR (Decapeptyl Trimestral[Title/Abstract])) OR (Trimestral, Decapeptyl[Title/Abstract])) OR (Decapeptyl LP[Title/Abstract])) OR (Decapeptyl Depot[Title/Abstract])) OR (gonadorelin agonist[Title/Abstract])) OR (GnRHa[Title/Abstract])) OR (GnRH agonist[Title/Abstract]) |
| #4 | ((((((((((((Chorionic Gonadotropin[MeSH Terms]) OR (Gonadotropin, Chorionic[Title/Abstract])) OR (Choriogonadotropin[Title/Abstract])) OR (Choriogonin[Title/Abstract])) OR (Pregnyl[Title/Abstract])) OR (Chorulon[Title/Abstract])) OR (Gonabion[Title/Abstract])) OR (biogonadyl[Title/Abstract])) OR (Chorionic Gonadotropin, Human[Title/Abstract])) OR (Gonadotropin, Human Chorionic[Title/Abstract])) OR (Human Chorionic Gonadotropin[Title/Abstract])) OR (HCG[Title/Abstract])) OR (Chorionic Gonadotropin[Title/Abstract]) |
| #5 | ((((((((((((((((Clinical Trial[MeSH Terms]) OR Controlled Clinical Trial[MeSH Terms]) OR Randomized Controlled Trial[MeSH Terms]) OR Intervention Study[Title/Abstract]) OR randomized trial[Title/Abstract]) OR trial, clinical[Title/Abstract]) OR clinical trial, controlled[Title/Abstract]) OR controlled clinical comparison[Title/Abstract]) OR controlled clinical drug trial[Title/Abstract]) OR controlled clinical experiment[Title/Abstract]) OR controlled clinical study[Title/Abstract]) OR controlled clinical test[Title/Abstract]) OR controlled trial, randomized[Title/Abstract]) OR randomised controlled study[Title/Abstract]) OR randomised controlled trial[Title/Abstract]) OR randomized controlled study[Title/Abstract]) OR trial, randomized controlled[Title/Abstract] |
| A total of 149 | (#1 OR #2) AND #3 AND #4 AND #5 |

Cochranel library

| #1 | MeSH descriptor: [Fertilization in Vitro] explode all trees OR (Fertilizations in Vitro):ti,ab,kw OR (Test-Tube Fertilizations):ti,ab,kw OR (Fertilization, Test-Tube):ti,ab,kw OR (Test Tube Fertilization):ti,ab,kw AND (Fertilizations, Test-Tube):ti,ab,kw OR (In Vitro Fertilizations):ti,ab,kw OR (Test-Tube Fertilization):ti,ab,kw OR (In Vitro Fertilization):ti,ab,kw OR (Test-Tube Babies):ti,ab,kw OR (Test Tube Babies):ti,ab,kw OR (Test-Tube Baby):ti,ab,kw OR (Baby, Test-Tube):ti,ab,kw |
| --- | --- |
| #2 | MeSH descriptor: [Sperm Injections, Intracytoplasmic] explode all trees OR (Sperm Injection, Intracytoplasmic):ti,ab,kw OR (Injection, Intracytoplasmic Sperm):ti,ab,kw OR (Intracytoplasmic Sperm Injection):ti,ab,kw OR (ICSI):ti,ab,kw OR (Injections, Sperm, Intracytoplasmic):ti,ab,kw OR (Intracytoplasmic Sperm Injections):ti,ab,kw OR (Injections, Intracytoplasmic Sperm):ti,ab,kw |
| #3 | MeSH descriptor: [Gonadotropin-Releasing Hormone] explode all trees OR (Gonadorelin Hydrochloride):ti,ab,kw OR (Gonadorelin Acetate):ti,ab,kw OR (Kryptocur):ti,ab,kw OR (Factrel):ti,ab,kw OR (Cystorelin):ti,ab,kw OR (Dirigestran):ti,ab,kw OR (FSH Releasing Hormone):ti,ab,kw OR (GnRH):ti,ab,kw OR (LH FSH Releasing Hormone):ti,ab,kw OR (Luliberin):ti,ab,kw OR (LH-RH):ti,ab,kw OR (Luteinizing Hormone Releasing Hormone):ti,ab,kw OR (Releasing Hormone, LHFSH):ti,ab,kw OR (LH FSH Releasing Hormone):ti,ab,kw OR (Gn-RH):ti,ab,kw OR (LH Releasing Hormone):ti,ab,kw OR (LHRH):ti,ab,kw OR (LHFSHRH):ti,ab,kw OR (LHFSH Releasing Hormone):ti,ab,kw OR (Gonadorelin):ti,ab,kw OR (FSH-Releasing Hormone):ti,ab,kw OR (LFRH):ti,ab,kw OR (LH-Releasing Hormone):ti,ab,kw OR (Luteinizing Hormone-Releasing Hormone):ti,ab,kw OR (Gonadoliberin):ti,ab,kw (LH-FSH Releasing Hormone):ti,ab,kw OR (Gonadotropin Releasing Hormone):ti,ab,kw |
| #4 | MeSH descriptor: [Chorionic Gonadotropin] explode all trees OR (Choriogonin):ti,ab,kw OR (Biogonadil):ti,ab,kw OR (Gonabion):ti,ab,kw OR (Pregnyl):ti,ab,kw OR (Choriogonadotropin):ti,ab,kw OR (Gonadotropin, Chorionic):ti,ab,kw OR (Chorulon):ti,ab,kw OR (HCG):ti,ab,kw OR (Human Chorionic Gonadotropin):ti,ab,kw OR (Chorionic Gonadotropin, Human):ti,ab,kw OR (Gonadotropin, Human Chorionic):ti,ab,kw |
| #5 | MeSH descriptor: [Clinical Trial] explode all trees OR MeSH descriptor: [Controlled Clinical Trial] explode all trees OR MeSH descriptor: [Randomized Controlled Trial] explode all trees OR (Intervention Study):ti,ab,kw OR (randomized trial):ti,ab,kw OR (controlled clinical drug trial):ti,ab,kw OR (controlled clinical comparison):ti,ab,kw OR (controlled clinical experiment):ti,ab,kw OR (controlled clinical study):ti,ab,kw OR (controlled clinical test):ti,ab,kw OR (randomised controlled study):ti,ab,kw OR (randomised controlled trial):ti,ab,kw OR (randomized controlled study):ti,ab,kw |
| A total of 594 | (#1 OR #2) AND #3 AND #4 AND #5 |

Embase

| #1 | 'intracytoplasmic sperm injection'/exp OR 'in vitro fertilization'/exp OR 'in vitro fertilization':ab,ti OR 'extracorporeal fertilization':ab,ti OR 'fertilization in vitro':ab,ti OR 'in vitro fertilisation':ab,ti OR ivf:ab,ti OR 'testtube baby':ab,ti OR icsi:ab,ti OR 'injection, intracytoplasmic sperm':ab,ti OR 'sperm injections':ab,ti OR intracytoplasmic:ab,ti |
| --- | --- |
| #2 | 'gonadorelin agonist'/exp OR 'cetrorelix'/exp OR 'gnrh agonist':ab,ti OR 'gonadotropin releasing hormone agonist':ab,ti OR 'LHRH agonist':ab,ti OR 'luteinising hormone releasing hormone agonist':ab,ti OR 'luteinizing hormone releasing hormone agonist':ab,ti OR 'GnRHa':ab,ti OR 'cetrorelix':ab,ti OR 'cetrorelix acetate':ab,ti OR 'cetrotide':ab,ti |
| #3 | 'chorionic gonadotropin'/exp OR 'chorionic gonadotropin':ab,ti OR 'gonadotropin, chorionic':ab,ti OR choriogonadotropin:ab,ti OR choriogonin:ab,ti OR pregnyl:ab,ti OR chorulon:ab,ti OR gonabion:ab,ti OR biogonadil:ab,ti OR 'chorionic gonadotropin, human':ab,ti OR 'gonadotropin, human chorionic':ab,ti OR 'human chorionic gonadotropin':ab,ti OR hcg:ab,ti |
| #4 | 'randomized controlled trial'/exp OR 'controlled clinical trial'/exp OR 'clinical trial'/exp OR 'Intervention Study':ab,ti OR 'randomized trial':ab,ti OR ' controlled clinical comparison':ab,ti OR 'controlled clinical drug trial':ab,ti OR 'controlled clinical experiment':ab,ti OR 'controlled clinical study':ab,ti OR 'controlled clinical test':ab,ti OR 'randomised controlled study':ab,ti OR 'randomised controlled trial':ab,ti OR ' randomized controlled study':ab,ti |
| A total of 911 | #1 AND #2 AND #3 AND #4 |

WOS

| #1 | TS= (fertilization in Vitro) OR TS=(In Vitro Fertilization) OR TS=(In Vitro Fertilizations) OR TS=(Test-Tube Fertilization) OR TS=(Fertilization, Test-Tube) OR TS=(Fertilizations, Test-Tube) OR TS=(Test Tube Fertilization) OR TS=(Test-Tube Fertilizations) OR TS=(Fertilizations in Vitro) OR TS= (Test-Tube Babies) OR TS=(Babies, Test-Tube) OR TS=(Baby, Test-Tube) OR TS=(Test Tube Babies) OR TS=(Test-Tube Baby) OR TS=(IVF) |
| --- | --- |
| #2 | TS=(Sperm Injections, Intracytoplasmic) OR TS=(Sperm Injections, Intracytoplasmic) OR TS=(Injection, Intracytoplasmic Sperm) OR TS=(Injections, Intracytoplasmic Sperm) OR TS=(Intracytoplasmic Sperm Injections) OR TS=(Sperm Injection, Intracytoplasmic) OR TS=(Intracytoplasmic Sperm Injections) OR TS=(ICSI) OR TS=(Injections, Sperm, Intracytoplasmic) |
| #3 | TS=(gonadorelin agonist)  OR  TS=(cetrorelix)  OR  TS=(gnrh agonist)  OR  TS=(gonadotropin releasing hormone agonist)  OR  TS=( LHRH agonist)  OR  TS=( luteinising hormone releasing hormone agonist)  OR  TS=( luteinizing hormone releasing hormone agonist)  OR  TS=(GnRHa)  OR  TS=(cetrorelix acetate)  OR  TS=(cetrotide) |
| #4 | TS=(Chorionic Gonadotropin)  OR  TS=(Gonadotropin, Chorionic)  OR  TS=(Choriogonadotropin)  OR  TS=(Choriogonin)  OR  TS=(Pregnyl)  OR  TS=(Chorulon)  OR  TS=(Gonabion)  OR  TS=(Biogonadil)  OR  TS=(Chorionic Gonadotropin, Human)  OR  TS=(Gonadotropin, Human Chorionic)  OR  TS=(Human Chorionic Gonadotropin)  OR  TS=(HCG)  OR  TS=(Chorionic Gonadotropin) |
| #5 | TS=(randomized controlled trial) OR TS=(controlled clinical trial) OR TS=(clinical trial) OR TS=(Intervention Study) OR TS=(randomized trial) OR TS=(controlled clinical comparison) OR TS=(controlled clinical drug trial) OR TS=(controlled clinical experiment) OR TS=(controlled clinical study) OR TS=(controlled clinical test) OR TS=(randomised controlled study) OR TS=(randomised controlled trial) OR TS=(randomized controlled study) |
| A total of 957 | (#1 OR #2) AND #3 AND #4 AND #5 |
